# Enrichment of genomic pathways based on differential DNA methylation profiles associated with knee osteoarthritis pain

**DOI:** 10.1016/j.ynpai.2022.100107

**Published:** 2022-11-03

**Authors:** Soamy Montesino-Goicolea, Lingsong Meng, Asha Rani, Zhiguang Huo, Thomas C. Foster, Roger B. Fillingim, Yenisel Cruz-Almeida

**Affiliations:** a Pain Research & Intervention Center of Excellence, University of Florida, Gainesville, FL, USA; b Center for Cognitive Aging & Memory, McKnight Brain Foundation, University of Florida, Gainesville, FL, USA; c Department of Biostatistics, College of Public Health & Health Professions and College of Medicine, University of Florida, Gainesville, FL, USA; d Department of Neuroscience, College of Medicine, University of Florida, Gainesville, FL, USA; e Institute on Aging, University of Florida, Gainesville, FL, USA; f Department of Community Dentistry & Behavioral Science, College of Dentistry, University of Florida, Gainesville, FL, USA

**Keywords:** Knee osteoarthritis, DNA methylation, Genomic pathways, Immune signaling, Older adult

## Abstract

Our study aimed to identify differentially methylated regions (i.e., genomic region where multiple adjacent CpG sites show differential methylation) and their enriched genomic pathways associated with knee osteoarthritis pain (KOA). We recruited cognitively healthy middle to older aged (age 45–85) adults with (n = 182) and without (n = 31) self-reported KOA pain. We also extracted DNA from peripheral blood that was analyzed using MethylationEPIC arrays. The R package *minfi* (Aryee et al., 2014) was used to perform methylation data preprocessing and quality control. To investigate biological pathways impacted by differential methylation, we performed pathway enrichment analysis using Ingenuity Pathway Analysis (IPA) to identify canonical pathways and upstream regulators. Annotated genes within ± 5 kb of the putative differentially methylated regions (DMRs, p < 0.05) were subjected to the IPA analysis. There was no significant difference in age, sex, study site between no pain and pain group (p > 0.05). Non-Hispanic black individuals were overrepresented in the pain group (p = 0.003). At raw p < 0.05 cutoff, we identified a total of 19,710 CpG probes, including 13,951 hypermethylated CpG probes, for which DNA methylation level was higher in the groups with highest pain grades. We also identified 5,759 hypomethylated CpG probes for which DNA methylation level was lower in the pain groups with higher pain grades. IPA revealed that pain-related DMRs were enriched across multiple pathways and upstream regulators. The top 10 canonical pathways were linked to cellular signaling processes related to immune responses (i.e., antigen presentation, PD-1, PD-L1 cancer immunotherapy, B cell development, IL-4 signaling, Th1 and Th2 activation pathway, and phagosome maturation). Moreover, in terms of upstream regulators, NDUFAF3 was the most significant (p = 8.6E-04) upstream regulator. Our findings support previous preliminary work suggesting the importance of epigenetic regulation of the immune system in knee pain and the need for future work to understand the epigenetic contributions to chronic pain.

## Highlights

• DNA methylation profiles differed between individuals with and without knee pain.
• IPA revealed that pain-related DMRs were enriched across multiple pathways and upstream regulators.
• Pathways and regulators were linked to cellular signaling processes related to immune responses.
• Current study findings replicate previous studies in persons with musculoskeletal pain.

## Introduction

Osteoarthritis (OA) is a disease mainly affecting hands, knees, and hips (Lubar et al., 2010) that is characterized by loss, degeneration, and calcification of articular cartilage Wu et al., 2020, i.e., “wear and tear” of joint cartilage (Zhang et al., 2019). OA is one of the most prevalent chronic joint diseases, with a higher prevalence in the older populations Zhang et al., 2019 and a significantly higher incidence in females than males (Nguyen et al., 2011). Notably, OA is a leading cause of chronic disability Blyth et al. (2019), Cross et al., (2014), a major public health concern and a significant economic burden. Despite this, there are no disease-modifying osteoarthritic drugs (DMOADs) approved by the US Food and Drug Administration (FDA) (Dunn et al., 2019). OA is a multifactorial disease (Reynard, 2017), with various genetic and non-genetic factors as key contributors to its development, including obesity, age, mechanical trauma, and local inflammatory processes Izda et al., 2021. In order to identify new therapeutic targets, we need to increase our understanding of the biological processes underlying OA development and progression. Genetic, transcriptomic, epigenetic, and proteomic analyses have all played crucial roles in identifying dysregulated pathways in OA (Reynard, (2017)). Specifically, the involvement of DNA methylation in OA pathophysiology has garnered considerable attention in recent years Rice et al., 2020, Sun et al., 2020, Zhou et al., 2020. DNA methylation process is the covalent addition of a methyl group to cytosine to form 5-methylcytosine under the catalysis of DNA methyltransferase (DNMT) (Wu et al., 2020). If applied to genes involved in OA-relevant biological processes, such changes in methylation status could accelerate the development of OA Miranda-Duarte, (2018). Thus, understanding drivers of DNA methylation in knee OA could provide the mechanistic knowledge needed for the development of advanced therapeutic strategies.

DNA methylation levels have been found to significantly differ between controls and individuals reporting low-back pain (Tajerian et al., 2011), neuropathic pain (Bell et al., 2014, Gombert et al., 2017, Sukenaga et al., 2016), chronic wide-spread musculoskeletal pain (Livshits et al., 2017, Bruehl et al., 2019, Burri et al., 2016) fibromyalgia in women Menzies et al., 2013, Ciampi De Andrade et al., 2017 and Crohn’s disease (Gombert et al., 2019, Li Yim et al., 2016). In OA, emerging literature links epigenetic changes in cartilage tissue, Zhao et al., 2017, Papathanasiou et al., 2019, subchondral bone (Jeffries et al., 2016, Zhang et al., 2016) and synovium (Yang et al., 2016, Zhang et al., 2020) to OA pathogenesis. In addition to these tissue-specific findings, a few studies (Dunn et al., 2019, Ebrahimi et al., 2020, Ai et al., 2021) report epigenetic changes in a peripheral blood sample in OA. For example, Dunn et al., 2019, investigated epigenetic patterns as models to predict future radiographic progression in OA patients, and suggested that peripheral blood mononuclear cells’ DNA methylation-based models may be useful as biomarkers of OA progression.

Our laboratory recently compared DNA methylation profiles between older adults with and without musculoskeletal pain (Montesino-Goicolea et al., 2020). Results provided preliminary insight into potential mechanistic changes at the cellular level associated with chronic musculoskeletal pain in older adults. Given that OA is one of the most common age-related musculoskeletal pain conditions, and based on previous work in OA, the goal of the present study was to identify differentially methylated CpG regions and their associated enriched genomic pathways in a larger, more racially diverse sample of middle-to older age adults with or at risk for knee osteoarthritis, using methods similar to those previously published by our group (Montesino-Goicolea et al., 2020). We hypothesized that DNA methylation profiles would be significantly different between participants with and without KOA pain and these would enrich different genomic pathways. To our knowledge, no study has examined DNA methylation differences among middle-older age adults with versus without chronic knee pain.

## Methods

The sample comprised community-dwelling adults recruited as part of an observational study at the University of Florida (UF) and the University of Alabama at Birmingham (UAB). Participants were aged 45–85 years who self-identified as non-Hispanic black (NHB) and non-Hispanic white (NHW), and reported unilateral or bilateral knee pain, and screened positive for clinical knee OA (Roux et al., 2008). Our lab's previous studies detailed a complete description of the recruitment, screening, exclusion, and inclusion criteria (Cruz-Almeida et al., 2017, Cardoso et al., 2016, Cruz-Almeida et al., 2013). In brief, participants were recruited through the community via multiple advertisement methods (e.g., posted fliers) and clinic-based methods. Potential participants were excluded if they reported 1) prosthetic knee replacement or other clinically significant surgery to the arthritic knee; 2) uncontrolled hypertension; 3) heart disease; 4) peripheral neuropathy in which pain testing was contraindicated; 5) systemic rheumatic disorders including rheumatoid arthritis, systemic lupus erythematosus, gout, and fibromyalgia; 6) neurological diseases such as Parkinson's, multiple sclerosis, stroke with loss of sensory or motor function, or uncontrolled seizures; 7) significantly greater pain in body sites other than the knee; 8) daily opioid use; 9) hospitalization within the preceding year for psychiatric illness, or 10) pregnant or nursing.

All procedures were reviewed and approved by the Institutional Review Boards of the UF and UAB, and all participants provided verbal and written informed consent. Participants came to the laboratory for completed a health assessment session (HAS) followed by a quantitative sensory testing session approximately-nine days apart as well as other visits, however, only measures relevant to the study hypotheses are included and presented below.

Participants were interviewed using a standardized pain history instrument regarding the presence of knee pain (e.g., history of knee surgery, knee pain duration, frequency of knee pain), and any other body regions where the individual experienced pain during the past three months (i.e., head/ face, neck, shoulders, arms, hands, chest, stomach, upper and lower back, leg, and feet). We also used the *Graded Chronic Pain Scale* as a widely validated and reliable tool adapted to assess characteristic knee pain intensity and pain-related disability specifically. Participants were asked to rate on a 0 (“no pain”) to 10 (“pain as bad as could be”) numerical rating scale their current knee pain, average knee pain, and worst knee pain in the past six months. These ratings were averaged and multiplied by 10 to yield a 0–100 score, with higher scores indicating more severe pain intensity. Pain-related disability (i.e., how much pain has interfered with daily activities, recreational/social/family activities, and ability to work) on average, over the past six months, was rated on a 0 (“no inference”) to 10 (“unable to carry out activities”) scale and multiplied by 10 to yield a 0–100 score, with higher scores indicating greater disability. Additionally, the GCPS asked respondents “How many days in the last six months have you been kept from your usual activities because of pain?” Disability points were calculated as the sum of the pain-related disability score (i.e., 0–29 = 0 points; 30–49 = 1 point; 50–69 = 2 points; ≥70 = 3 points), and total number of disability days (i.e., 0–6 days = 0 points; 7–14 days = 1 point; 15–30 days = 2 points; 31 days or more = 3 points). Scores from the GCPS characteristic pain intensity scale and Disability points were then used to categorize participants according to a pain grade: Grade 0 = no reported pain intensity; Grade 1 = low disability (i.e., <3 disability points) and low pain intensity (i.e., <50); Grade 2 = low disability-high intensity pain (i.e., ≥50); Grade 3 = high disability-moderately limiting (i.e., 3–4 Disability Points), regardless of pain intensity; Grade 4 = high disability-severely limiting (i.e., 5–6 Disability Points), regardless of pain intensity.

### Blood collection and processing

Blood samples were collected from the forearm or hand vein at the onset of the second study visit and included collection of a 10 ml K2 EDTA tube and a 7 ml Corvac Serum Separator tube that were subsequently used for DNA methylation and Vitamin D analyses, respectively.

### DNA extraction and methylation analysis

The EDTA tube was centrifuged at 3000 rpm for 10 min and the buffy coat was carefully extracted and transferred to a cryovial for −80-degree storage. To isolate genomic DNA, the frozen buffy coat samples were thawed at 37 °C to dissolve homogeneously. ∼ 200 ul (or 150–200 ul) of sample was lysed in R.B.C lysis buffer and centrifuged at 6000 rpm for 5 min at room temperature. The supernatant was discarded and sodium EDTA solution was added to the pellet and vortex gently to remove RBC clumps. Homogenate was incubated at 50–55 °C with Proteinase K and SDS solution. Following incubation, equal volume of phenol was added, mixed, and centrifuged at 10,000 rpm for 10 min. Supernatant was transferred in a fresh tube and equal volume of phenol–chloroform-isoamyl alcohol was added, mixed and centrifuged at the same rpm. Again, supernatant was transferred in a fresh tube and equal volume of chloroform-isoamyl alcohol was added followed by centrifugation at same rpm conditions. Supernatant was transferred in a fresh tube and 1/10th volume of 3 M sodium acetate along with 2 volumes of absolute alcohol was added. The precipitated DNA was washed with 70 % ethanol by centrifugation at 10,000 rpm for 5 min. The pellet was air dried and dissolved in Tris-EDTA buffer. The dissolved DNA was qubit quantified and visualized on agarose gel for quality assessment. Sodium Bisulfite conversion and EPIC methylation array was performed by Moffitt Cancer Center, Molecular Genomics Core located at 3011 Holly Dr., Tampa, FL 33612.

### DNA methylation data preprocessing

R package *minfi* (Ritchie et al., 2015) was used to perform methylation data preprocessing and quality control. Illumina Human Methylation EPIC array annotation (hg19) was employed for genome mapping. Functional normalization was applied to perform between-array normalization and regress out variability explained by the control probes. Among all 866,091 CpG probes, we removed (1) 1,275 probes with non-significant detection p-value (p > 0.01) in>10 % samples; (2) 30,064 probes containing a single nucleotide polymorphism (SNP) either at the CpG interrogation or at the single nucleotide extension; and (3) 18,920 probes on the sex chromosome. Totally, 815,633 CpG probes remained in our final analysis.

### Differentially methylated probes (DMPs)/ differentially methylated regions (DMRs) associated with pain

To identify pain-related DMPs, we applied the linear model approach followed by the empirical Bayes moderated *t*-statistics test implemented in the *limma* package (Jaffe et al., 2012). In this model, the methylation level of a CpG probe was the outcome variable, the pain grade was the predictor, adjusting for age, sex, race, and study site as a covariate. Since adjacent CpG probes are highly correlated and pain-related CpG probes could be clustered into genomic regions, we further performed region-based differential methylation analysis using R *bumphunter* package (Benjamini and Hochberg, 1995), which can perform genomic segmentation, create CpG clusters, identify DMRs (i.e., genomic region where multiple adjacent CpG sites show differential methylation) using a similar linear model approach, and obtain the statistical significance of a DMR via permutation test. Multiple testing was adjusted using the false discovery rate (FDR) (Lawrence et al., 2013).

### Functional annotation by genomic features

To explore the potential functional effect of pain-related DMPs on transcriptional activities, we annotated the putative DMPs to genomic features using the R package (Lawrence et al., 2013) *GenomicFeatures*, including promoters, exons, introns, and intergenic regions.

### Pathway enrichment analysis

To investigate the pathway activities about differential methylation, we performed pathway analysis using Ingenuity Pathway Analysis (IPA) to identify canonical pathways and upstream regulators. Gene duplicates were removed before performing the analysis. Annotated genes within ± 5 kb of the putative DMRs (p < 0.05) were subject to the IPA analysis. For consistency with our previous work, IPA analysis was not separated into hypo- and hyper-methylated DMRs and the study was only powered for aggregated analysis.

## Results

### Demographics

Our study included 213 participants between 44 and 78 years old, the mean age was 57.7 (±7.9), and 129 (60.6 %) were female. Table 1 shows the detailed demographics stratified by pain and disability groups (i.e., No pain, pain-low disability and pain-high disability) using the GCPS (n = 213). There was no significant difference in age, sex, study site among pain groups (p > 0.05). Non-Hispanic black individuals were overrepresented in the pain group (p = 0.003).

**Table 1:** Characteristics of the study participants.

	GCPS Grade 0 No pain (n = 31)	GCPS Grades 1–2 Pain-Low Disability (n = 107)	GCPS Grades 3–4 Pain-High Disability (n = 75)	
Age, Mean (SD)	58.6 (9.2)	58.6 (7.7)	56.3 (7.3)	0.125
Sex, No. (%)				0.770
Male	12 (38.7)	40 (37.4)	32 (42.7)	
Female	19 (61.3)	67 (62.6)	43 (57.3)	
Race, No. (%)				**0.003**
Non-Hispanic black	12 (38.7)	41 (38.3)	47 (62.7)	
Non-Hispanic white	19 (61.3)	66 (61.7)	28 (37.3)	
Study site, No. (%)				0.212
University of Florida	18 (58.1)	73 (68.2)	42 (56.0)	
University of Alabama at Birmingham	13 (41.9)	34 (31.8)	33 (44.0)	

*: The p-values were obtained by ANOVA for continuous variable, or $\chi^{2}$-test for categorical variables.

### DMPs/DMRs associated with pain

In terms of DMPs, at FDR-adjusted p < 0.05, we did not identify any significant differentially methylated CpG probes. At raw p < 0.05 cutoff, we identified total 19,710 CpG probes, including 13,951 hypermethylated CpG probes (i.e., DNA methylation level is higher in the group with higher pain grade) and 5,759 hypomethylated CpG probes (i.e., DNA methylation level is lower in the group with higher pain grade). The top 20 DMPs are shown in Table 2, and the full list is shown in Table S1. The heatmap of the top putative DMPs is shown in Fig. S1.

**Table 2:** Top 20 differentially methylated probes (DMPs).

CpG probe	Chr	Start	End	Feature	Direction*	p value	Genes†
cg16475306	15	71,348,075	71,348,075	intergenic	–	2.24E-06	
cg13477812	4	186,435,506	186,435,506	introns	+	2.90E-06	PDLIM3
cg00743372	6	28,109,648	28,109,648	promoters	–	4.15E-06	ZSCAN16-AS1;ZKSCAN8
cg18980580	2	20,440,781	20,440,781	intergenic	+	8.24E-06	
cg09749703	2	153,192,460	153,192,460	promoters	–	1.05E-05	FMNL2
cg17880816	19	3,789,435	3,789,435	introns	+	1.98E-05	MATK
cg05441107	10	134,972,886	134,972,886	intergenic	+	3.26E-05	KNDC1
cg15801215	6	130,341,195	130,341,195	introns	–	3.97E-05	L3MBTL3
cg19733255	9	74,918,970	74,918,970	intergenic	+	4.21E-05	
cg26144070	21	45,874,614	45,874,614	promoters	+	4.28E-05	LRRC3-DT;LRRC3
cg00119127	7	1,422,999	1,422,999	intergenic	+	4.29E-05	
cg03883941	11	33,384,058	33,384,058	intergenic	–	5.20E-05	
cg03486986	13	112,986,285	112,986,285	intergenic	+	5.22E-05	
cg08936253	18	44,334,486	44,334,486	introns	+	5.50E-05	ST8SIA5
cg06843871	2	242,740,810	242,740,810	introns	+	8.66E-05	GAL3ST2
cg01438216	3	28,070,171	28,070,171	intergenic	–	9.30E-05	
cg13969581	19	5,620,148	5,620,148	introns	+	1.13E-04	SAFB2;SAFB
cg08765940	11	133,789,708	133,789,708	exons	+	1.16E-04	IGSF9B
cg17723381	12	76,114,755	76,114,755	intergenic	+	1.16E-04	
cg10310847	7	6,048,008	6,048,008	promoters	–	1.25E-04	PMS2;AIMP2

*: + indicates hypermethylation (higher methylation level in the pain group as compared to the no-pain group); and - indicates hypomethylation (lower methylation level in the pain group as compared to the no-pain group).

†: Annotated genes within ± 5 kb of the CpG probe.

In terms of DMRs, all CpG probes can be clustered into 3,863 regions, among which 4 regions are differentially methylated at FDR-adjusted p < 0.05 cutoff, including 2 hypermethylated regions and 2 hypomethylated regions. At raw p < 0.05, 557 regions are differentially methylated, including 397 hypermethylated regions and 160 hypomethylated regions. The top 20 DMRs are shown in Table 3, and the full list is shown in Table S2.

**Table 3:** Top 20 differentially methylated regions (DMRs).

Chr	Start	End	Direction*	p value	q value	Genes†
6	30,039,132	30,039,524	–	1.56E-05	0.023	PPP1R11;RNF39
6	29,648,161	29,648,628	–	1.61E-05	0.023	ZFP57
5	1,594,330	1,594,863	+	1.79E-05	0.023	SDHAP3;LOC728613
8	216,578	216,788	+	2.55E-05	0.025	
11	17,791,761	17,791,761	+	8.04E-05	0.057	KCNC1
8	7,320,532	7,320,532	–	9.90E-05	0.057	SPAG11B
11	134,608,598	134,608,598	+	1.03E-04	0.057	
14	24,779,959	24,780,825	+	1.27E-04	0.057	CIDEB;LTB4R2;LTB4R
12	130,821,962	130,822,605	+	1.55E-04	0.057	PIWIL1
9	124,989,294	124,990,276	+	1.81E-04	0.057	LHX6
14	33,407,059	33,407,370	+	2.00E-04	0.057	NPAS3
8	58,055,591	58,056,175	+	2.10E-04	0.057	
1	202,172,778	202,172,912	+	2.25E-04	0.057	LGR6
7	4,244,250	4,244,643	+	2.32E-04	0.057	SDK1
17	78,231,489	78,231,489	–	2.45E-04	0.057	SLC26A11;RNF213
2	105,275,601	105,276,153	+	2.51E-04	0.057	
8	2,585,666	2,586,008	+	2.69E-04	0.057	
8	599,963	600,233	–	2.77E-04	0.057	
5	171,057,072	171,057,339	+	2.95E-04	0.057	
11	128,693,961	128,694,679	+	3.13E-04	0.057	

* : + indicates hypermethylation (higher methylation level in the pain group as compared to the no-pain group); and - indicates hypomethylation (lower methylation level in the pain group as compared to the no-pain group).

† : Annotated genes within ± 5 kb of the CpG probe.

### Functional annotation by genomic features

**Fig. 1:**
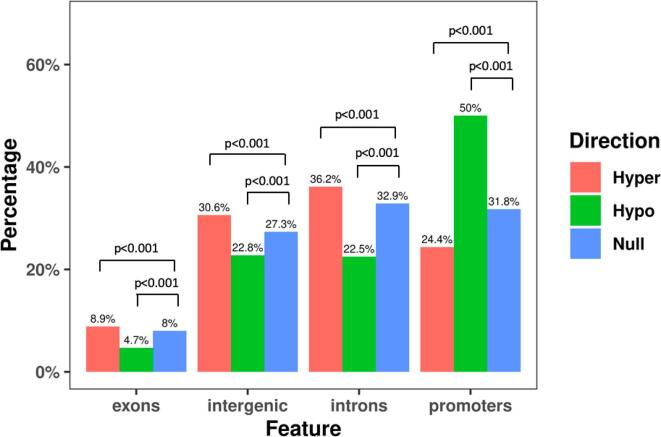
Genomic feature distributions of all putative DMPs (raw p < 0.05). ***(color must be used for this printed figure)***. (For interpretation of the references to color in this figure legend, the reader is referred to the web version of this article.)

To examine the potential functional effect of pain-related DMPs on transcriptional activities, we annotated the putative DMPs (raw p < 0.05) to predetermined genomic features (Fig. 1). Compared to the null distribution of CpG probes included in the Illumina EPIC array, hypermethylated probes were enriched in introns (36.2 % vs 32.9 %), intergenic probes (30.6 % vs 27.3 %), and exons (8.9 % vs 8 %) but depleted in promoters (24.4 % vs 31.8 %,). By contrast, hypomethylated probes were most enriched in promoters (50 % vs 31.8 %), but depleted in exons (4.7 % vs 8 %), intergenic probes (22.8 % vs 27.3 %), and introns (22.5 % vs 32.9 %). All these contrasts were statistically significant (p < 0.001).

### Enrichment analysis

**Fig. 2:**
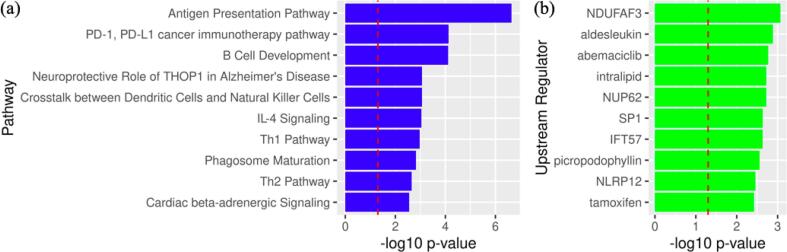
Pathway enrichment analysis using Ingenuity Pathway Analysis: (a) result for top 10 canonical pathways and (b) result for top 10 upstream regulators. The vertical dashed red line indicates the p = 0.05 level. ***(color must be used for this printed figure)***. (For interpretation of the references to color in this figure legend, the reader is referred to the web version of this article.)

Pathway enrichment analysis revealed that the pain-related DMRs (raw p < 0.05) were enriched across multiple canonical pathways and upstream regulators (see details in Table S3 and Table S4**)**. In terms of the canonical pathway, the antigen presentation pathway was the most significant (p = 1.31E-03), followed by PD-1, PD-L1 cancer immunotherapy pathway (p = 1.62E-02) and B cell development, (p = 1.64E-02). In terms of upstream regulator, NDUFAF3 was the most significant (p = 8.6E-04). The top 10 canonical pathways are shown in Fig. 2**a**, and the top 10 upstream regulators are shown in Fig. 2**b**.

## Discussion

The present study evaluated DNA methylation profile associations with knee OA-related pain in a large sample of middle-to-older-aged adults. We employed an integrative computational analysis to identify targetable pathways enriched by the genes with differentially methylated CpG sites. At present, despite intense research, our understanding of the pathogenesis of knee OA-related pain remains limited, and thus, treatments primarily aim to relieve symptoms. Microarray and sequencing technology have been widely used to identify potential therapeutic targets for OA pain. Understanding the molecular mechanisms of knee OA pain can provide information to enhance diagnosis and treatment. Interestingly, the top DMRs/DMPs were not annotated to any pain-relevant genes, but were mainly reflective of cellular responses important for immune signaling as discussed below.

The majority of the ten pathways identified in this study are related directly to the innate and adaptative immune systems (i.e., the antigen presentation pathway, B cell development, IL-4 signaling, Th1 pathway, Th2 pathway, phagosome maduration, and, crosstalk between dendritic cells, and natural killer cells) confirming the critical role of the immune system in symptomatic knee OA pain. Our findings are consistent with a recent DNA methylation study where a significant hypomethylation of inflammation-related genes correlated with an increased expression of pro-inflammatory cytokines in patients with hip OA (Rushton et al., 2015). The literature has shown that macrophages, T cells, mast cells, B cells, plasma cells, natural killer cells, dendritic cells, and granulocytes have been identified in the synovial membranes of OA patients (Saito et al., 2002, de Lange-Brokaar et al., 2012, Da et al., 2007, Benito et al., 2005, Nakano et al., 2007, Pessler et al., 2008). Among them, macrophages and T cells most abundantly infiltrate the synovial tissues of OA patients, representing approximately 65 % and 22 %, respectively (Symons et al., 1991). Also, patients with OA have shown higher levels of CD4 in the peripheral blood and the synovial fluid compared with healthy controls, which suggests that Th cells in both serum and the synovial fluid may be involved in the pathogenesis of OA (Fields et al., 2019). Overall, there is enough evidence supporting an increased number of genes related to cytokine production and inflammatory/defense response in knee OA participants.

Further, the antigen presentation pathway is the initial part of the dynamics of the immune response, and the cytokine secretion is one of the effector responses. IL-1β, a pro-inflammatory cytokine (Boraschi et al., 2018) that is expressed on chondrocytes, synoviocytes, osteoblasts, osteoclasts, and inflammatory cells such as macrophages in the knee joint (Jenei-Lanzl et al., 2019), induces catabolic events such as cartilage degradation via mitogen-activated protein kinase (MAPK) signaling (Molnar et al., 2021);(Silvestri et al., 2006). On the other hand, elevated levels of soluble IL-4 receptor have been observed in the serum of OA patients (Yeh et al., 1995), and an IL-4/IL-10 system was recently demonstrated to reduce expression of matrix metalloproteinases and induce proteoglycan synthesis in vivo, which have a chondroprotective effect (Doi et al., 2008, Chen et al., 2017). Overall, this suggests that the epigenetic regulation of cytokine synthesis is an exciting area to be explored and could offer new solutions in OA pain management.

The present pathway enrichment analysis findings are very similar to those reported in previous work from our lab (Montesino-Goicolea et al., 2020). In both studies, the antigen presentation pathway and the PD-1, PD-L1 cancer immunotherapy pathway resulted the most significant among B cell development, Th1 and Th2 activation pathway, and IL-4 signaling. These findings seem to be in agreement with the literature; even though PD-1/PD-L1 signaling has been mainly targeted for cancer immunotherapy, it may also serve as an endogenous pain inhibitor and a neuromodulator (Canavan et al., 2021). In addition, previous studies have confirmed a significant increase in synovial PD-1 expression in early and established persons with rheumatoid arthritis (RA) compared to healthy controls and OA synovial tissue (Canavan et al., 2021). Moreover, Raptopoulou et al. (Raptopoulou et al., 2010) (Freeman et al., 2000) confirmed that the histological expression of PD-1 correlates with the degree of synovial inflammation; also, PD-1/PD-L1 reduces the secretion of pro-inflammatory cytokines, such as IFN-γ, TNF-α, IL-2, IL-6, and IL-3 to inhibit T cell activation (Rushton et al., 2014). Further studies are needed to investigate the role of the PD-1/PD-L1 axis within the microenvironment in a knee OA joint.

In a gene ontology analysis, Rushton and colleagues (2014) (Juhas et al., 2015) revealed an enrichment of genes involved in the immune response and inflammation, including IL-2, IL-3, IL-4, and IL-6, as well as several differentially methylated loci in genes involved in TGF‐β and in cartilage degradation and homeostasis. Our results seem to show the same pattern; for example, the macrophage pathway. The activation of macrophages and initiation of the activation of transcriptional mechanisms also leads to the release of cytokines, chemokines, and growth factors (Southam et al., 2007). Among the growth factors, recent studies have highlighted the contributions of TGF- β1 to OA pathogenesis (Reynard et al., 2011). Also, members of the transforming growth factor‐β (TGF‐β) superfamily are involved in the development, maintenance, and repair of bone, cartilage, and other soft tissues of the synovial joint. Our results show a higher methylation of GDF7 with increasing pain and compared to the no-pain group, but there are no DNA methylation studies to date that directly involve GDF7 in persons with KOA. However, aberrant methylation at the promoter and 5′‐UTR locations of another member of the TGF- β superfamily (GDF5), has been reported in OA patients (Chidambaran et al., 2019). The GDF5 gene repression, resulting from the aberrant methylation (Rushton et al., 2015), causes a poorly developed cartilage with a lack of efficient regeneration. Future studies should consider epigenetic mechanisms of macrophage activation in this target population.

The current study has some limitations. First, our sample included individuals with knee OA symptoms but did not require radiographic confirmation of OA. Thus, our findings may be more relevant to symptomatology rather than OA pathophysiology. Second, our analysis was based on whole blood samples and not specific to tissue in the nervous system related to pain processing (e.g., brain, DRG) or to cartilaginous and bone tissue related to OA disease (e.g. cartilage, chondrocytes and bone). Third, variations in blood cell composition may affect the results of the methylation analysis, although, in other pain studies, this was not observed. Fourth, it is not currently known whether the observed epigenetic patterns cause or are a consequence of chronic pain in our participants. Fifth, there is a lack of validation and inclusion of all DMRs irrespective of the level of methylation differences; and how much methylation difference between groups is significant enough to interfere with gene expression remains unclear. Finally, we used computational analyses to evaluate the pathways associated with epigenetic group differences but did not examine gene or protein expression levels. Given the complexity and multiple levels of gene regulation, future more extensive studies are needed to evaluate gene regulation using epigenetics (i.e., considering hypo- versus hyper-methylation) and actual gene and protein expression levels concerning knee OA pain.

In summary, the current study provides a better understanding of the role of epigenetic regulation in symptomatic knee OA. Given the extensive literature already documenting the interaction of immune cells with nociceptors in preclinical and clinical studies, the study of underlying epigenetic mechanisms driving pain can have important clinical and therapeutic implications and should be taken into consideration when developing therapeutic targets. Although future studies are required in larger prospective cohorts with longitudinal evaluation of various epigenetic measures, these results are promising, and open new avenues for epigenetics-based pain research.

## Funding

This work was supported by 10.13039/100000002NIH/NIA Grants R01AG067757 (YCA); and R37AG033906 (RBF). A portion of this work was performed in the McKnight Brain Institute at the National High Magnetic Field Laboratory’s Advanced Magnetic Resonance Imaging and Spectroscopy (AMRIS) Facility, which is supported by 10.13039/100000001National Science Foundation Cooperative Agreement No. DMR-1157490 and DMR-1644779 and the State of Florida.

## Declaration of Competing Interest

The authors declare that they have no known competing financial interests or personal relationships that could have appeared to influence the work reported in this paper.
