# Supplementary material for: Enrichment of genomic pathways based on differential DNA methylation profiles associated with knee osteoarthritis pain
Source: Neurobiol Pain. 2022 Nov 3;12:100107. doi: 10.1016/j.ynpai.2022.100107 (PMC9755025; doi:10.1016/j.ynpai.2022.100107)

Figure S1. Heatmap visualization of the very significant putative DMPs (p < 0.005). The color key indicates the z-score of the methylation value. The colors red and blue indicate higher and lower methylation value, respectively. Black and orange color bar on top of the heatmap indicates no-pain and pain groups, respectively. ***(color must be used for this printed figure)***


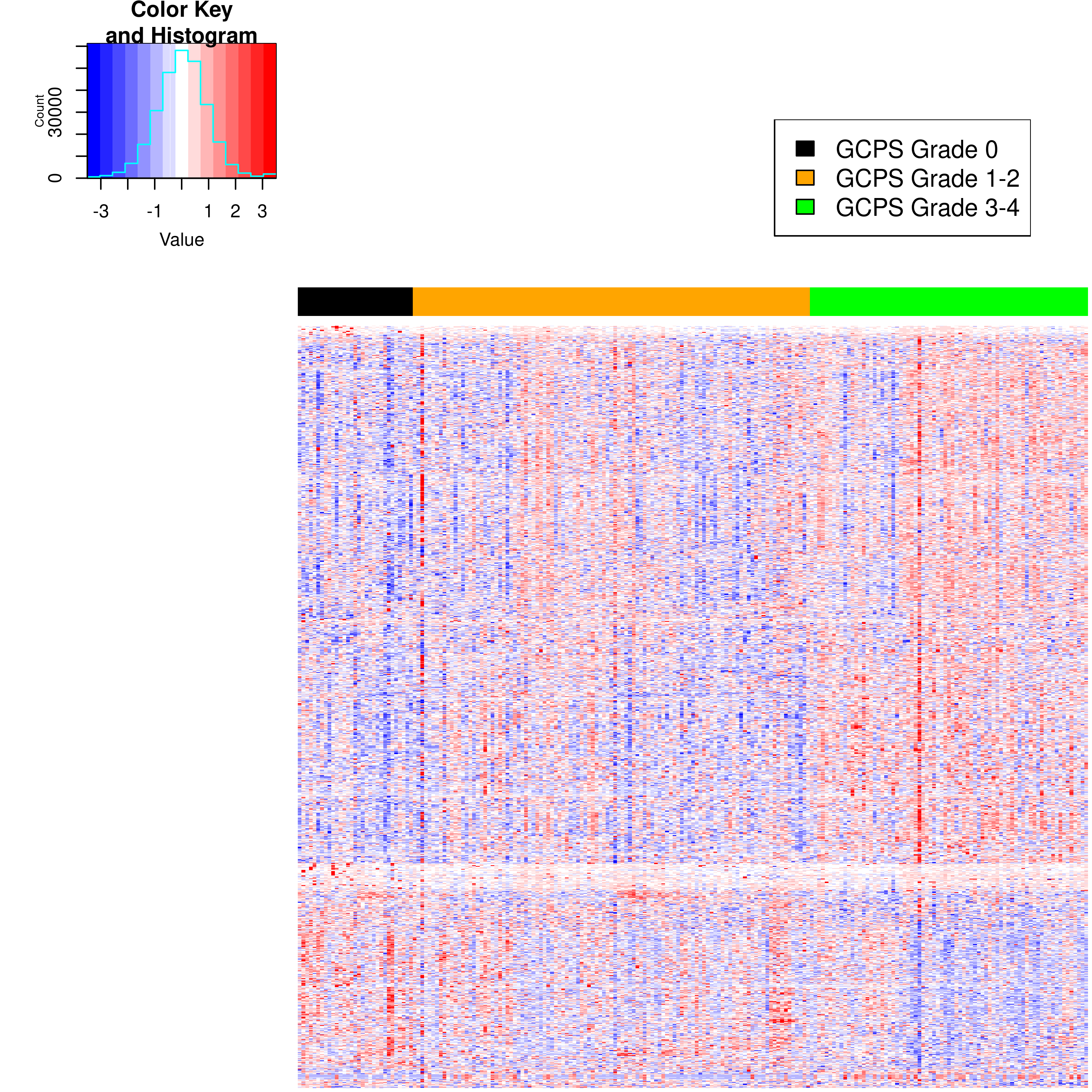

Supplement: Supplementary data 5 [file mmc5.docx]
